# Patients’ perception regarding the influence of individual and social vulnerabilities on the adherence to tuberculosis treatment: a qualitative study

**DOI:** 10.1186/s12889-017-4752-3

**Published:** 2017-09-19

**Authors:** Rosiane Davina da Silva, Fernanda Darliane Tavares de Luna, Aguinaldo José de Araújo, Edwirde Luiz Silva Camêlo, Maria Rita Bertolozzi, Paula Hino, Sheylla Nadjane Batista Lacerda, Sayonara Maria Lia Fook, Tânia Maria Ribeiro Monteiro de Figueiredo

**Affiliations:** 1 Universidade Estadual da Paraíba, Campina Grande, Paraíba Brazil; 2 Universidade Federal do Rio Grande do Norte, Natal, Rio Grande do Norte Brazil; 3 Escola de Enfermagem da Universidade de São Paulo, São Paulo, São Paulo Brazil; 4 Escola Paulista de Enfermagem da Universidade Federal de São Paulo, São Paulo, Brazil; 5 Faculdade Santa Maria, Cajazeiras, Paraíba Brazil

**Keywords:** Tuberculosis, Vulnerability, Adherence to medication

## Abstract

**Background:**

Tuberculosis remains an important disease which mainly affects the majority of vulnerable individuals in society, who are subjected to poor living conditions and difficulties to access the services of public health. Under these circumstances, the present study aims to understand patients’ perception in relation to the influence of individual and social vulnerabilities on the adherence to tuberculosis treatment.

**Methods:**

A qualitative descriptive cross sectional study was conducted in one large municipality at the state of Paraíba, Northeast of Brazil. The study subjects, who were residents of the study site, covered all tuberculosis cases diagnosed between March and June 2015. The sample was defined by the criteria of response saturation. All interviews were audio recorded, and data analysis was developed through the hermeneutic dialectic method and the theory of Generative Route Sense. The project was approved by the Research Ethics Committee of the University of São Paulo (USP).

**Results:**

A total of 13 individuals were interviewed and the responses were identified into two analytical categories: the difficulties they had and the enabling factors they could mention during their tuberculosis treatment. Patients brought up social exclusion as an obstacle to treatment adherence, which, along with stigmatization, weakened their link with family members and health professionals. Moreover, economic precariousness was a major hindrance to the maintenance of a proper diet and transportation access to health centers. However, social support and directly observed treatment helped to break down barriers of prejudice and to promote individual and family empowerment. Finally, patients also reported that their will to live and faith gave them the strength to continue with the treatment.

**Conclusions:**

According to patients in this study, social support and the strengthening of links with family members and health professionals may reduce social exclusion and other difficulties they face, thus encouraging them to the adhere to tuberculosis treatment.

## Background

Tuberculosis (TB) remains as one of the major global infectious diseases. It is estimated that one third of the world’s population is infected by the *Mycobacterium tuberculosis* and that 10, 4 million individuals were affected by the disease in 2015 [1].

The World Health Organization prioritized a list of 30 countries that concentrate 87.1% of the total global burden of TB cases [1]. According to international ranking indicators, Brazil takes the 20^th^ position on this list. In fact, a total of 63.189 cases of the disease were reported in the country in 2015, including pulmonary and extrapulmonary forms, which corresponds to an incidence of 33 cases per 100.000 population. In the state of Paraíba, 1.525 cases of TB were registered, with an incidence rate of 40.5 cases per 100,000 in habitants [2] and 79 death cases [3].

The WHO established the following goals for the control of the disease for the year 2015: to halt and reverse TB incidence coefficient according to what was proposed by the Millennium Development Goals, and to reduce prevalence and mortality indicators by 50% in relation to the 90s according to the Stop TB Partnership strategy [4]. Brazil achieved the goals related to incidence, prevalence and mortality [5]. However, cure and loss to follow-up rates are still below those proposed by the WHO and agreed by the National TB Control Program, which are to cure at least 85% of the cases and to reduce loss to follow up to levels below 5% [1, 6]. In the year 2014, 75.1% of the patients were cured and 11.3% individuals were lost to follow up in the country. In the state of Paraíba, the cure and loss to follow-up rates were respectively 65.5% and 11.8% [2]. This is an alarming situation since the low cure and the high loss to follow-up rates can lead to an increase in the TB incidence and mortality levels all over the country [7].

In this context, treatment adherence plays a central role in the disease control given the fact the treatment favors improvement in the indicators of loss to follow up and cure [8]. Nevertheless, disease control remains as a challenge for global public policies; treatment adherence is a multicausal process that transcends biological, clinical or behavioral aspects, and it is associated with the way patients conceive the disease, their social condition that should allow for the development of life with dignity, and the support from health services. Adherence is, in fact, influenced by the social determinants of the health-disease process [9].

Many are the barriers that increase vulnerability and the risk for treatment non-adherence. Among these barriers are the long duration of treatment, drug intolerance associated with clinical improvement at the initial months of treatment [8], low socioeconomic and school level, lack of knowledge about the disease, lack of social incentives, homeless living situations, alcoholism and illicit drug use [10, 11], social and individual stigma and lack of support from family members and health professionals [12–14].

There is a predominance of the disease among male patients in the economically active age group. This group is considered vulnerable to treatment loss to follow-up [15] since many times patients’ working hours and health services opening hours are incompatible, thus making the access to such services very difficult [16]. Additionally, the reduction of work capacity due to the disease itself and the adverse effects of the antituberculosis drugs [8, 12] contributes to treatment non-adherence.

Treatment non-adherence contributes to higher rates of treatment loss to follow-up and multidrug resistance [1]. These are factors that hinder the cure process, bring suffering and cause economic losses not only to the patient, who is frequently laid off from work, but also to the state budget since the treatment becomes more costly [8, 17].

Therefore, whenever socioeconomic diseases like TB are studied, treatment adherence must be studied under the light of vulnerability [18]. In a broader sense, vulnerability reflects the potential for getting sick, for not getting sick and for coping with the health-disease process resultant from the interconnected and inseparable individual, social and programmatic aspects. The potential for getting sick reflects the social inequity that affects the general population, which, in turn, stimulates (or not) the adherence to treatment [19].

The individual vulnerability is determined by cognitive conditions inherent to the access to the information on the health-disease process and the capacity to elaborate and put this information into practice on a daily basis. In this context, the individual holds certain rights that must be protected by the social aspect insofar as the sociopolitical, economic and cultural context can favor the access to social resources, paramount to the maintenance of health, and the adoption of social protection measures. Such measures should be reinforced by the programmatic plan that is based on the political and institutional commitment to the funding, operationalization and evaluation of public programs and policies for disease prevention as well as health promotion and assistance [19, 20].

Hence, in order to identify the vulnerabilities in the process of treatment adherence and establish a strategy for effective interventions, not only individual elements (behavioral and clinical) should be taken into consideration but also the relation of the subjects to their social contexts, their life conditions and accessibility to the available disease control programs and services [19]. Such aspects are strongly related to the individual, social and programmatic vulnerabilities.

Although the three aspects of vulnerability are inseparable, Ayres, Paiva, França et al. [20] state that for a deeper understanding of vulnerability, it must be considered that the social environment plays an important role in individual behavior, and in this dimension, relations between people and the world are established [21]. Therefore, it is important to keep these factors of individual and social vulnerability in perspective, in order to identify the potentialities and fragilities in patients in their fight against the disease, so that strategies that favor their adherence to treatment can be strengthened and the loss to follow-up rate can be reduced.

The present study aim to understand patients’ perception in relation to the influence of individual and social vulnerabilities on the adherence to tuberculosis treatment. As a result, it may shed some light on the importance of the operationalization of intersectoral and social assistance actions that will help managers, health authority and society to see the individual as a whole in the social environment.

## Methods

### Study setting

**Fig. 1:**
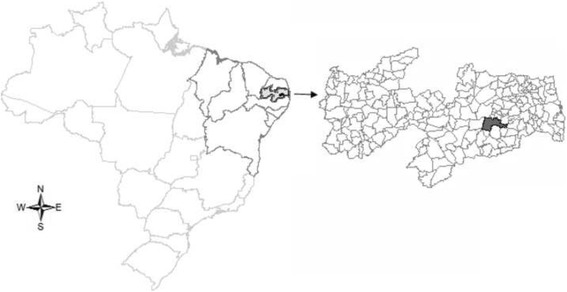
Brazil, Northeast region, state of Paraiba and municipality of Campina Grande

The location adopted for this study was the municipality of Campina Grande, (Fig. 1), located in the mesoregion of Agreste Paraibano, Northeast of Brazil. Considered the second largest municipality in the state of Paraiba in terms of territorial extension (407.754 km2), population level (593.026 inhabitants) and economic importance. The human development index (HDI) in the municipality is of 0.720 [22].

The municipality has an important university center and attracts students from other states of Brazil, which contributes to the increase of the local economy. The main economic activities developed in the municipality include agriculture, livestock, mineral extraction, handicrafts, trade. It also stands out in the production of software for export, and for this reason is considered one of the main industrial hub of the Northeast Region and the largest technological hub of Latin America. It has the second largest GDP in the state, accounting for 15.63% of the total wealth produced in Paraíba [22].

In relation to health sector, the municipality has a structured network of health services which is a reference for other municipalities in Paraíba and Brazilian states. According to the state’s geopolitical administrative division, the municipality is home to the third Regional Health Center covering 70 municipalities of the state [23].

The municipality was chosen for this study because it concentrates the second largest tuberculosis burden in the state, and is considered a priority by the National Tuberculosis Control Program, TB control actions are developed by 94 family health teams, distributed in 88 Basic Health Units [24], by one Secondary Outpatient Clinic in TB, with support from the tertiary level by the municipal hospitals and the Clementino Fraga Hospital, a reference related to patients with infectious diseases, located in the capital of State (João Pessoa) 130 km away from the municipality.

### Study design

This is a multicentric, qualitative, descriptive, cross-sectional study that evaluated the effectiveness of social protection measures towards the improvement of programmatic indicators for TB treatment adherence and control of the disease in some regions of Brazil.

The studied population comprised all cases of tuberculosis diagnosed between March and June, 2015, totaling 45 patients. The screening process included under-treatment patients aged 18 years or older, who were capable of understanding and answering questions, and lived in the area where the study was conducted. Prison inmates or hospitalized patients were excluded, remaining 20 individuals to be contacted.

For the definitive composition of the sample, it was requested the database of the National System of Notifiable Diseases of the Municipal Health Department of the municipality studied, from which a list with the name, telephone number and address of the patients was extracted, then the subjects were contacted in a simple random manner, by randomness and interviewed according to their availability. The sample was defined based on the saturation of the information contained in the statements.

Data were collected in August 2015, at the place of choice of the patients, domicile or health service, through an interview with a semi-structured script. The initial questions gathered data about the characterization of the subjects: sex, age, level of schooling and clinical form of tuberculosis. The following steps were questions like: What difficulties and facilities did you have during the tuberculosis treatment? Depending on the subject’s responses, the researcher formulated new questions for monitoring, allowing a deeper understanding of the subject and observing the individual perceptions of tuberculosis treatment, allowing the participants to guide the discussion.

Researchers were coached on the conduction of the interview prior to data collection. The interview lasted around 20 min, and the statements were audio recorded and fully transcribed^1^ doubly by two researchers, by isolation form, on a Microsoft Word 2010 document. The studied subjects were then identified by the letter I, which stands for the word interviewee, and numbered according to the order of the interview (I1, I2, I3, and so on).

The responses were analyzed in pairs and then were interpreted the light of dialectical hermeneutics [25] and the theory of Generative Route Sense [26]. The integration of the two analysis techniques aims to achieve a broader complementarity so that a more realistic view of the studied context could be assessed; thus, the knowledge, interpretation and reinterpretation [27] of the patient’s history in the process of adhering to the TB treatment within a social background can be better understood.

Hermeneutics deals with the analysis of texts, statements and narratives. Its interpretation is not detached from praxis, and it is intrinsically related to intersubjectivity, namely the ability to put oneself in someone else’s place. It takes the individual’s discourse into consideration within the historical and totalizing specificity in which it is produced. Dialectic tries to discuss contradictory facts in language, signs and culture; the dialectical process suggests that the interpretative analysis should emphasize social phenomena according to the dynamicity they occur. The connection of these concepts allows for the comprehension of how facts express themselves and occur from the interaction between individuals and the understanding of the reality expressed in the discourse [25].

The theory of Generative Route Sense is based on the understanding that language and discourse cannot be dissociated from the individual’s view of the world in accordance with life conditions and social relations. Therefore, the discourse reveals the reality and enables the understanding of the text by the identification of figures that are expressed by words or concrete expressions. When interrelated, they form a figurative pathway; the sense of this narrative set is represented by a central theme of the discourse related to abstract elements that organize and categorize the senses as well as the articulation between figures and themes. As a result, the restructuring of the discourses into theme sentences occur, reflecting the perception of individuals as to the objective of the study [26].

### Human ethics approval

The study was approved by the Research Ethics Committee from the School of Nursing at the University of São Paulo (USP) under the number 37254714.0.1001.5392.

## Results and discussion

The characteristics of the 13 interviewed patients are shown in Table 1. There was a prevalence of males (76.9%) between 21 and 35 years of age (59.9%), with 4 to 7 years of schooling (46.2%) and pulmonary form of TB (92.3%).

**Table 1:** Overall characteristics of the studied patients

Characterization of the Studied Individuals			
		No	%
Sex	Male	10	76.9
	Female	3	23.1
Age Group	21–25	3	23.1
	26–30	1	7.7
	31–35	3	23.1
	36–40	1	7.7
	41–45	0	─
	46–50	2	15,3
	51–55	1	7.7
	56–60	0	─
	>60	2	15.3
Educational Level	Unschooled	1	7.7
	1–3 years	2	15.3
	4–7 years	6	46.2
	8–11 years	3	23.1
	>12 years	1	7.7
Clinical Form	Pulmonary	12	92,3
	Extra-pulmonary	1	7,7

After the statements were analyzed, they were divided into two analytical categories: the difficulties they had and the enabling factors they could mention during their tuberculosis treatment, based on individual and social vulnerability elements.

**Category 1**: Difficulties met during the treatment.

According to the participants, “*Many were the difficulties*” (I1, I2, I4, I5, I11, I12, I13) to be overcome during the treatment. The first obstacles were “*prejudice*” (I5, I11 and I13) and social discrimination: “*(…) you are condemned for having the disease”* (I12), especially by family members and friends; *“I’ve been very criticized, extremely judged (…*). *People didn’t want to help me in any way.”* (I13).

As can be noted, TB still carries stigmas and prejudices that are harmful to the *“TB guy”* (I12). The suffering the disease brings is worsened with the segregation and social isolation. For patients, the idea of sharing the diagnosis with family and friends resided in the fact that they needed to find the strength to fight the disease; however, some were caught by surprise when they started being avoided by those people around them, which reinforces the prejudicial perception and makes the individual vulnerable to the non-adhesion to treatment.

In fear of this kind of reaction, some patients decide to *“hide the disease”* (I12, I11) as a strategy to keep a social life. Not revealing the diagnosis is thus a solution they find to avoid rejection and shame as well as an obstacle to treatment at the same time*: “It’s tough for the guy to explain (...) it’s embarrassing (...)”* (I11). Furthermore, patients’ self-stigmatization regarding the perception of the disease supported by ideologies and current social values may trigger an existential crisis and a low self-esteem process: *“How would people see me? As a TB guy or as a human being?”* (I12).

Therefore, the negative attributions to TB patients make acceptance and control of the disease difficult. Patients feel unmotivated to undergo the treatment and the evaluation of TB contacts by health professionals is hindered, causing a delay in the detection of latent and active TB cases [28].

According to Lacerda et al. [17], social stigma comes from the general population’s lack of knowledge on TB. Thus, health education plays an important role in the explanation of the disease and treatment to patients and family members, allowing for the deconstruction of stigmas and prejudices [29]. It is essential that health professionals see beyond the biomedical model, which focuses on the disease and the individual, and have a more intersubjective, dialogical approach so that patients with their knowledge can be taken into consideration. In such case, the aim is not only to inform patients about the shades of the health-disease process but also to change their concepts through the development of health awareness [30, 31].

In result, directly observed treatment (DOT) is an indispensible tool for the therapy, once it aims to monitor the medication intake and strengthen the bond of health professionals with patients and family members. DOT brings them closer to health services and provides full assistance in biological and psychosocial terms, which allows for the previous detection of vulnerability aspects that may interfere with treatment adherence [32]. Therefore, it is of utmost importance that health professionals pay careful heed to patients’ needs.

All the care and support supplied to patients by health services help to strengthen the bond and the co-responsibility in the treatment process [27]. On the other hand, a weak link tends to cripple the cure process: *“The doctor is a pain in the neck; she doesn’t treat us right; she is very rude”* (I5). Such situation raises vulnerability issues regarding the non-adherence to treatment given the fact the patient feels unmotivated to search for assistance in health centers.

According to the statements, it was possible to infer that patients perceived the lack of social support and the self-administered treatment model as barriers to start therapy, especially when they had other comorbidities like depression, for example: *“As I suffer from depression (...) sometimes I forget to take the medicine (...) then it starts all over again”* (I13). Vulnerability to the non-adherence is again increased since the patient feels discouraged to face the disease alone, particularly when drug interactions and the adverse effects of the antituberculosis drugs occur [32, 33].

As for antituberculosis drugs, patients stated that *“the medication is strong”* (I4), *“tastes awful, especially in the first months of treatment”* (I2), when they are more debilitated and the medication is more powerful (Coxcip- 4^2^), with adverse effects that directly interfere with their daily habits: *“It's hell really; it feels like I’m gonna faint, and sometimes I don’t sleep at night”* (I1). This situation, along with specific symptoms of the disease - *“pain in the lungs”* (I5), *“the person gets tired and sweats a lot”* (I11) - directly impose limitations to patients in their labor activities (I5, I6, I13). Medical leaves of absence are frequent – *“I stopped working for 3 months ‘cause I couldn’t be exposed to the cold weather. Since I work at night, things got messed up,”* (I3) - and even by unemployment too (I1, I4, I11, I12).

The situation is aggravated when the patient is a male within the economically active age group, which is the prevailing profile of this study. Historically, these men are in charge of providing financial support to their families; thus, incapacity for work results in socioeconomic losses not only for patients, but also for their dependents. In the very first months, the false sensation of health recovery, associated with the discomfort caused by the adverse effects of the drugs, lead these patients to treatment non-adherence due to the urge to go back to work.

Work incapacity negatively interferes with the family budget. Many patients could not afford *“the well-balance diet”* (I4, I5, I6, I12, I13) required by the treatment: *“Food that we didn’t have”* (I1), *“One day I barely had something to eat”* (I4), “*If only I had some juice to take the medicine (...), but i’s hard to swallow it down with water because it tastes like kerosene.”* (I6).

Besides weakening the immune system and affecting patients’ clinical evolution [34], malnutrition causes enhanced adverse effects like nausea, weakness and abdominal pain [35]. It is clear that adverse effects and the economic situation form a vicious cycle, in which one condition may trigger the other.

Poor living conditions make accessibility to health services even more difficult when health centers are far from the patient’s residence. In such cases, the bond between health professionals and patients is affected (I1, I4, I5, I12), and treatment costs become significantly higher when transportation expenses are added to the family budget, especially in the face of the *“current economic crisis the country has been going through,”* (I5) and the lack of community support and social incentives: *“You are a structure without any financial support; you have nothing.”* (I12).

The fact that patients who live far from health centers have to commute to undergo the DOT may be a hindrance to treatment continuity, either due to the lack of financial support or the time spent on transportation [36]. According to Luna et al. [16], long distances also weaken the execution of programmatic strategies, like home visits by health professionals. However, other studies reveal that in some situations the farther the services are from home, the easier it is for the patient to adhere to the treatment. In other words, the health center is seen as a haven from exclusion and neighborhood discrimination [11, 37]. In this study, some patients also mentioned the distance as a convenience, namely a form of protection against prejudice.

Such finding shows that no matter how near or how far patients live from health services, their perceptions regarding this distance vary from difficult to convenient according to the social context they are in. It is implied then that the decentralization of TB control actions to the primary care level will only be successful when society overcomes the stigma of the disease and understands that the cooperation from all citizens is essential for TB control. Nevertheless, while the desired outcome is not achieved, in-home DOT expansion strategies should be designed and financial support for transportation to and from health services should be provided. These temporary solutions would help not only to strengthen adherence to treatment but also to reduce social exclusion.

In the literature, the distribution of incentives (“baskets” of basic food staples, snacks, transportation vouchers and disability benefits) have proven to be very important for the continuation of treatment and the reduction of social exclusion [33], so such incentives should be provided to patients during the therapeutic period. However, it was observed that most of the patients under treatment did not receive any kind of food or financial support, probably because of the lack of funds of the PNCT from the studied municipality. Consequently, patients feel discouraged to adhere to the treatment, especially those who are more economically disadvantaged.

In a study conducted in Argentina, the authors reveal that patients undergoing financial hardship who had to pay for transportation costs to and from health services were approximately three times more likely to abandon the treatment than those who received transportation vouchers [36]. In Brazil, many studies show that incentives like food and financial support strengthen treatment adherence [11, 37].

Alcoholism was also mentioned as a barrier to treatment adherence: *“I ended up drinking, and I knew that I couldn’t drink alcohol under treatment, so I didn't take the medicine”* (I9). It is known that chronic alcoholism makes patients susceptible to malnutrition and immunodepression, which delay the cure process and intensify adverse reactions [17, 38]. Moreover, this group suffers from social exclusion and emotional disorders, including lack of family support [39], which interfere with the disease acceptance and put patients in a situation of vulnerability to treatment non-adherence.

**Category 2**: Enabling factors during TB treatment.

According to patients, the distribution of antituberculosis drugs by the Public Health Network helps adherence, but by itself it is not enough for treatment continuity. First of all, therapeutic adherence is boosted by the will of living and the desire to be healed, particularly when patients are motivated to develop life projects and they are bound by ties of affection.

Achieving success in TB treatment becomes easier when patients keep social relations flowing and do not suffer from the stigma. Interviewees reported that support from *“family members”* (I5, I8, I10, I11) and *“some friends”* (I1) *“motivated them to move on”* (I11) and *“continue with the treatment”* (I5, I8, I10, I11). Furthermore, this support helped them accept the disease and overcome obstacles.

The family represents the social group closest to patients, whose support is fundamental to treatment adherence. Therefore, health professionals can not only provide a more qualified assistance targeting patients’ needs but also develop health educational actions on individual and family empowerment once they know the family dynamics [40].

According to the perception of patients, health professionals’ support was also fundamental to treatment continuity (I1, I11, I13): *“The doctor told me to continue with the treatment so that I could heal”* (I11); “*Once I made up my mind to stop taking the medicine; then the nurse came, insisted (...). I spent two weeks without taking the medicine, and after the nurse and the social assistant strongly insisted, I started taking it again.”* (I13).

In this context, the strengthened bond helped establish a trust relationship, based on dialogue and respect, between patients, family members and health professionals, which favored the understanding of the importance of the treatment and its correct application [15].

DOT has proven to be a valuable tool for adherence once it gives health professionals a better understanding of patients’ perceptions on the fight against the disease in relation to the social context they are in. As a result, a broader and more qualified assistance can be provided to patients according to their needs, as seen in other studies [16, 29]: *“It’s good (...) The orderlies come and clearly explain everything to me.”* (I8).

The proximity of patients’ residences to health services was also a determining factor for treatment continuity and DOT performance: *“Because the health center is very close to my house, I don’t have to pay for transportation. But if I had to go every day (...) and pay the bus fare to take the medicine, then it would be expensive”* (I9). This situation can compensate the lack of social incentives and the low-income situation of local patients [41].

Faith and religiosity also took on an important role in treatment continuity. Patients feel strong enough to cope with all the difficulties imposed by the disease, and they believe that in the hope of *“feeling better”* (I7), their recovery occurs through intervention of the divine. The energy acquired though faith leverages their cure desire, and substantially contributes to treatment adherence [8, 11] and the return to daily activities like work [33].

Upon analyzing the responses regarding the enabling factors and difficulties in the disease treatment, it was possible to observe that patients perceive social exclusion as the greatest difficulty for treatment adherence. This exclusion is sometimes intensified by stigmatization/auto-stigmatization, segregation/social isolation, financial issues or lack of social protection incentives, or eased by the strengthening of the bond with family members and health professionals. Moreover, according to patients, faith and religiosity, when linked to the will to live, positively influenced on the decision to continue with the treatment and be cured.

The study also revealed the urgency of combating stigma, which may perhaps be obtained through Health education strategies that highlight the patient-family engagement, and target the individual and collective empowerment regarding the deconstruction of prejudices, the strengthening of interpersonal bonds and a greater political awareness in the fight for health rights. The directly observed treatment (DOT) program also needs to be expanded, and social protection strategies, like food and financial support, must be improved so that the social exclusion issue is minimized.

As possible limitations of the study, the punctual period of data collection can be pointed out, in which resulted in a reduced sample size and whose discourses may have suffered interference from the historical, political and temporal context in the study site.

## Conclusions

The difficulties and enabling factors reported by the TB patients reinforce the view that only the availability and free distribution of the medication is not enough for treatment continuity or the cure. There are social and individual vulnerability aspects that may hinder or strengthen this process. These findings were also highlighted in other cultural realities distributed in other regions and countries of the world, based on literature review, which brings about new insights and understanding about the health of the population.

Therefore, it is noticeable that the TB issue involves multiple conditioning factors. Its control is complex due to the fact it demands reciprocal moral and ethical commitment from the whole society to patients, who, as social actors, must struggle for dignified life conditions.

The epidemiological control of the disease is broad, and it has to go through barriers from political government agencies. It is questionable whether global efforts for the control of the disease are enough for the care of the individual as a whole, or whether they are targeted to the achievement of very specific quantitative goals, which may or may not be accomplished, depending on the socio-historical context in which the individual is inserted.

From this perspective, it is necessary to strengthen the role of primary health care in the assistance provided to these individuals for the identification of vulnerabilities and reduction of health inequities in order to ensure that care actions will be carried out in a bidirectional and reciprocal way. It will then be possible to see patients beyond the disease and encourage them to move on with their life projects so that these patients can contribute to the potential adherence and success of the treatment. Moreover, these care actions should be reinforced by social incentives, especially at municipal level, geographically responsible for the execution of disease action control.

It is important that health professionals and managers recognize tuberculosis as a socially determined disease. They should also try to understand the social context in which the patient is inserted and the patient’s perception on the factors connected to the disease, in such a way that treatment adherence can be strengthened and cure and loss-to-follow-up rates can be improved with the active and critical participation of patients and the society.

## Abbreviations

(DOT): Directly observed treatment
(GDP): Gross domestic product
(HDI): Human development index
(PNCT): National tuberculosis control program
(SINAN): National system of notifiable diseases
(TB): Tuberculosis
(WHO): World health organization

## Footnotes

1 The responses were accurately translated into the English language in the final phase of the writing manuscript preparation.

2 Coxcip-4 is a drug in pill form with fixed-dose combination of rifampicin, isoniazid, pyrazinamide and ethambutol. It is used in the acute phase of the disease (the first 2 months of the antituberculosis treatment)^4^.
